# Antimutagenic and anticancer activity of Darjeeling tea in multiple test systems

**DOI:** 10.1186/1472-6882-14-327

**Published:** 2014-09-02

**Authors:** Udayan Bhattacharya, Shanta Adak, Niladri Shekhar Majumder, Biswajit Bera, Ashok K Giri

**Affiliations:** Molecular and Human Genetics Division, Indian Institute of Chemical Biology, 4, Raja S. C. Mullick Road, Jadavpur, 700 032 Kolkata India; Department of Zoology, Seth Anandram Jaipuria College, Kolkata, India; Tea Board, Kolkata, India

**Keywords:** Darjeeling tea, Antimutagenic, Anticancer, Apoptosis

## Abstract

**Background:**

Darjeeling tea, a most popular variety of black tea, though consumed by the people in different parts of world but its beneficial health effects have not been investigated in details. In this study, the antimutagenic and anticancer effect of Darjeeling tea extract (DTE) has been evaluated.

**Methods:**

Antimutagenic activity of the DTE was carried out in two different strains of *Salmonella typhimurium* by AMES test against a known mutagen benzo[a]pyrene (B[a]P) with S9 activation. Moreover, anticlastogenic property of DTE was also measured by micronuclei formation (MN) against B[a]P with S9 activation in human lymphocytes. The anticancer activity of the same was studied on U937 cell line. Here, Human PBMCs were used as the normal cell control to identify selective anticancer activity of the extract against U937 cells.

**Results:**

The results showed significant antimutagenic activity on bacterial strains. A significant decrease in MN was also observed in the DTE treated human lymphocyte cultures pretreated with B[a]P when compared with B[a]P treated cultures alone. The study clearly exhibited anticancer activity of the extract on U937 cell line. Further studies also revealed that apoptosis induction is an important mechanism behind the anticancer effect of DTE.

**Conclusion:**

Overall, this study indicates that DTE has significant antimutagenic and anticancer activities on bacterial and mammalian cells respectively.

## Background

Tea, made from the tender shoots (two leaves and a bud) of the plant *Camellia sinensis* L. is the second most popular beverage of the world after water. According to its processing, tea can be classified into few categories of which two types are most common viz. green tea and black tea. Since last few decades tea has received a great deal of attention due to their beneficial health effects [1]. It has been found that tea is rich in substances, such as polyphenols, which are capable of reducing the risk of a variety of human illnesses, including cancer or uncontrolled cell proliferation [2]. It is known that transition of a normal cell to a cancer cell is a multi-step process which is triggered by genetic mutations.

Epidemiological and laboratory studies have indicated that regular consumption of tea has been associated with reduced risk of several forms of cancer in human and also in mouse experimental model [3, 4]. Scientific studies have also revealed that tea is able to reduce the DNA mutation rate. It is known that tea extracts have antimutagenic [5–8] and anticancer activities [9–17]. Though Black tea is more common than green tea, until recently, tea research related to human health, has mostly been focused on green tea. However, recent studies indicate that black tea can also provide health benefits similar to that of green tea [18].

Darjeeling teas are the highest grown teas in the world in terms of altitude and preferred for its flavour, aroma and quality throughout the world from centuries [19]. It is also considered to be one of the most delicate kinds of tea worldwide. But, there is hardly any report on its antimutagenic and anticancer activities to date. Therefore, this study was aimed to investigate the antimutagenic and anticancer potential of Darjeeling tea extract (DTE) by different standard laboratory methods.

## Methods

### Materials

Darjeeling tea (cultivated at 2800 m of elevation) sample was kindly provided by Tea Board, Kolkata, India. Agar and nutrient broth were purchased from HiMedia laboratories Ltd (India). RPMI 1640 medium liquid from Hyclone (Utah, USA), penicillin, and streptomycin were obtained from Pan Biotech (Aidenbach, Germany), gentamycin from Cambrex Bio Science Walkersville Inc (MD, USA), cell proliferation _reagent_ WST-1 [2-(4-Iodophenyl)-3-(4-nitrophenyl)-5-(2,4-disulfophenyl)-2H-tetrazolium] was obtained from Roche Diagnostics (Indianapolis, Indiana, USA), Alexa Fluor 488 annexin V/Dead Cell Apoptosis Kit with Alexa Fluor 488 annexin V and PI for Flow Cytometry, fetal bovine serum (FBS), phytohemagglutinin (M form) and amphotericin (B from) were purchased from Invitrogen (Carlsbad, CA, USA), caspase protease assay kit was purchased from Chemicon International Corporation (Temecula, CA, USA), Mitochondria/Cytosol fractionation kit from BioVision (Mountain View, CA, USA), Primary antibody of cytochrome c and β-actin and polyclonal secondary antibody were obtained from Cell Signaling Technology (Danvers, MA, USA). The Bio-Rad Protein Assay Kit was from Bio-Rad Laboratories (Hercules, CA, USA). Biotin, histidine, nicotinamide adenine dinucleotide phosphate (NADP), glucose-6- phosphate, ampicillin trihydrate, tetracycline, cytochalasin B, benzo[a]pyrene (B[a]P), dimethyl sulphoxide (DMSO), Dithiothreitol (DTT), colchicines, Tween-20, NBT/BCIP, absolute ethanol, isopropanol, agarose, and tris-acetate buffer were purchased from from Sigma-Aldrich Co. (St. Louis, MO, USA).

### Bacterial strains

For antimutagenicity assays, *Salmonella* strains TA98 and TA100 were used. These strains were kindly provided by Dr. Bruce N. Ames, Biochemistry Division, University of California, Berkeley, USA.

### Animal

Charles River male rats of 150–175 g. were used for the preparation of liver homogenate (S9) for bacterial antimutagenicity assays. Four animals were kept per cage with husk bedding which were received from the animal house of our institute and were fed balanced rodent pellet diet (Gold Mohar, Lipton Ltd., Chandigarh, India) and water. The animals were kept in an environment with controlled 12 h light and 12 h dark cycle. Ambient temperature and relative humidity were kept 22° ± 2°C and 55% ± 5% respectively. All experimental procedures on animals were approved by the Institutional Animal Ethics Committee of CSIR-Indian Institute of Chemical Biology.

### Preparation of S9 fraction

The procedure of Ames et al. [20] and Garner et al. [21] was used for the preparation of rat liver homogenate (S9). Charles river male rats of 150–175 g were fed 0.1% phenobarbital in their drinking water for seven days. On day 6, no foods were provided for these rats. The next day, they were killed for the rat liver homogenate. S9 mix was prepared following the method of Maron and Ames [22]. All steps were performed at 0° to 4°C with cold and sterile solutions and glassware. S9 fractions were distributed in 2 ml aliquots in small sterile plastic tubes, quickly frozen and stored at -80°C.

### Preparation of Darjeeling tea extract (DTE)

Darjeeling tea (100 g) was brewed in boiling water (1000 ml) for 2 minutes and filtered. Then the extract was evaporated to dryness by rotatory evaporator to yield around 8 g of whole tea extract denoted as DTE.

### Mutagenicity and antimutagenicity assays in preincubation tests

Standard mutagenicity assays in preincubation tests were performed for both mutagenicity and antimutagenicity assays [22]. DTE was dissolved in DMSO and different concentrations of DTE (1, 10, 100, 500, 1000 μg/plate) were used in both mutagenicity assay and antimutagenicity assay against known mutagen B[a]P. A similar experiment was carried out with B[a]P (50 μg/plate) alone, with metabolic activation (S9 mix), which served as positive control. For preincubation tests bacteria, different concentrations of DTE, positive compound B[a]P (50 μg/plate) and phosphate buffer were co-incubated in the S9 mix for 20 min at 37°C and then top agar was added to it. Within 1 h the plates were inverted and placed in a dark, vented incubator at 37°C for 48 h. Four plates were used for each concentrations tested and for both positive and negative controls.

Finally, the revertant colonies on the test plates were counted. The experiment was repeated twice with similar set up. All these experiments were carried out using liver homogenate (S9) fractions for both the strains. Presence of background lawn on all the plates was confirmed. The spontaneous reversion rates of these two different *Salmonella* strains were checked and those were similar as reported by other authors.

### Micronucleus assay (MN) in vitro in human lymphocytes

Blood samples were collected from six healthy individuals (3 male and 3 female) and for MN analysis lymphocytes were cultured following the protocol of Fenech [23]. For this work, Human Ethical committee permission was taken from the Human Ethical Committee of CSIR-Indian Institute of Chemical Biology to collect human blood samples and all work was done according the approved protocol. Consent was obtained from each human subjects for the use of their blood in research. After collection, whole blood (0.7 ml) was added to 7 ml of RPMI 1640 supplemented with L-glutamine, 15% FCS, penicillin (100 IU/ml), streptomycin (100 μg/ml) and 2% phytohemaglutinin M-form. From each blood samples ten cultures were carried out. After the 24 h of initial incubation B[*a*]P (100 μM) plus S9 mix were added to four cultures from each samples and then incubated for 3 h [24]. The cultures were then washed twice with RPMI medium containing 1% FCS. Then four concentrations (25, 50, 100, 200 μg/ml) of DTE were dissolved in DMSO and were added to the above mentioned cultures. After initial 24 h of incubation, one culture from each sample was treated with only B[*a*]P and S9 mix which served as positive control. One culture from each sample was also treated with DMSO plus S9 mix only which will serve as negative control at the same time point. Other remaining cultures were treated with different concentrations (25, 50, 100, 200 μg/ml) of DTE only which also serve as DTE treated control at the same time in parallel. Then all the ten cultures from each sample were further incubated for another 20 h and then cytochalasin B was added to each culture at the final concentration 6 μg/ml and the cultures were incubated for an additional 28 h. After a total of 72 h of incubation, the cells were centrifuged at approximately 1000 rpm for 5 min. Supernatant was discarded and cell pellets were treated with a hypotonic solution. Cells were mixed with freshly prepared methanol: acetic acid (3:1) for 10 min and then centrifuged at approximately 1000 rpm for 5 min. After air drying the slides were stained with 4% Giemsa in phosphate buffer, pH 7.3, for 10 min. At least 2000 cytokinesis-blocked binucleated human lymphocytes with preserved cytoplasm were scored whenever possible from each of the six donors for all the concentrations tested. MNs were identified in accordance with the criteria followed by Fenech et al. [25].

### Cell culture

U937 (Human Histiocytic Lymphoma) cell was obtained from National Centre for Cell Science (NCCS), Pune, India. The cells were cultured in RPMI 1640 medium, supplemented with 10% heat inactivated and sterile fetal bovine serum (FBS), penicillin (100 units/ml), streptomycin (100 μg/ml), gentamycin (100 μg/ml) and amphotericin B (1.2 mg/ml) and incubated at 37°C in a humidified atmosphere containing 5% CO_2_ inside a CO_2_ incubator.

### Isolation of PBMCs (Peripheral blood mononuclear cells)

PBMCs were isolated from heparinized whole blood of normal human by density gradient centrifugation on Histopaque. Briefly, fresh heparinized blood was mixed with equal volume of PBS. 10 ml of diluted blood was gently layered on 5 ml of Histopaque in a 15 ml conical tube and centrifuged at 2000 rpm at 4°C for 40 minutes with no brake. Cells were collected from the white interphase between the plasma and transparent fraction and resuspended in RPMI-1640 with 10% heat-inactivated fetal bovine serum and maintained at 37°C in a humidified atmosphere containing 5% CO_2_ inside an incubator for 2 hours on cell culture dishes. After that non-adherent cells were washed out gently and the rest of the PBMCs were used in cell viability assay as normal cells.

### Cell viability assay using WST-1

PBMCs and U937 cells were obtained from culture medium and loaded in a haemocytometer for cell count before plating of the cells. 10^5^ cells were plated per well in 96 well plates and treated with DTE. Four concentrations (25, 50, 100, 200 μg/ml) of DTE including a control (0.05% DMSO) were administered in triplicate. Campothecin B (5 μM, final concentration) was used as a positive control to check the assay was functioning correctly. The plates were incubated for 24 h, 48 h and 72 h respectively at 37°C in a humidified atmosphere containing 5% CO_2_. Cell viability was measured using WST-1 following manufacturer’s protocol. Briefly, 10 μl of WST-1 reagent was added to each well and incubated for 3 h. Absorbance was measured at 450 nm using an ELISA reader (Beckman Coulter, CA, USA) with a reference serving as blank. The viability of control cells were taken as 100% and were measured from the absorbance value. The viability of treated cells were calculated similarly from the absorbance values and expressed as percentage of control viability.

### Detection of apoptosis by flow cytometry

U937 cells (1X10^6^ in each case) were treated with different concentrations (25, 50, 100 μg/ml) of DTE for 48 hours along with positive and negative controls as mentioned in the previous section. Apoptosis was measured using AnnexinV-PI Apoptosis Detection Kit. U937 cells were harvested and PI and Annexin V conjugated fluos were added to the medium and then analyzed on flow-cytometer (Becton Dickinson, San Diego, CA; with 488 nm argon laser light source; 530 nm band pass filter for Alexa fluor 488-fluorescence and 623 nm band pass filter for PI- fluorescence) using CellQuest software. A total of 10,000 events were acquired and the cells were properly gated for analysis.

### Detection of apoptosis by confocal microscopy

U937 cells (10^6^ cells/ml) were cultured in a 60-mm culture dishes initially for 24 h and then treated with 0, 25, 50, and 100 μg/ml doses of DTE for 48 h along with the respective controls. The cells were treated with DAPI for labeling the nuclei, followed by incubation with FITC-AnnexinV to detect phosphatidylserine at the outer surface of cell membrane. Stained nuclei and phophatidylserine were visualized and photographed using a Nikon A1R laser scanning confocal microscope (Nikon Inc, Melville, New York, USA). DAPI (excitation at 350 nm and emission at 470 nm) and Fluorescein Isothiocyanate (FITC) (excitation at 490 nm and emission at 525 nm) were excited by the argon–krypton laser. Apoptosis was characterized by detecting externalization of phosphatidylserine.

### Measuring activity of caspase-3

Activity of Caspase-3 was assayed using caspase-3 fluoremetric assay kit. U937 cells (2×10^6^) were treated with three different concentrations of DTE (0, 25, 50, 100 μg/ml) for 48 h and the activity was determined fluorimetrically (excitation, 400 nm and emission 505 nm).

### Preparation of cytosolic fraction and western blot analysis of cytochrome c

U937 cells were harvested after treatment with 0, 25, 50 and 100 μg/ml concentrations of DTE for 48 h. Isolation of a highly enriched mitochondrial fraction and cytosolic fraction of cells was performed using a Mitochondria/Cytosol fractionation kit. Briefly, U937 cells (5 × 10^7^) were centrifuged at 600 g for 5 min at 4°C, resuspended in ice-cold PBS and centrifuged at 600 g for 5 min at 4°C. Then the cells were resuspended in 1.0 ml of cytosol extraction buffer mix containing DTT and protease inhibitors and incubated on ice for 10 min. The cells were homogenized on ice. The homogenate was centrifuged at 700 g for 10 min at 4°C and the supernatant was collected and centrifuged at 10,000 g for 30 min at 4°C. Then, the supernatant was collected as the cytosolic fraction. This isolated cytosolic fraction was subjected to western blot analysis for cytochrome c release assay. Cytochrome c expression was analyzed with anti-cytochrome c polyclonal antibody. An alkaline phosphatase-conjugated goat anti-rabbit secondary antibody was used for this experiment. For western blot analysis the protein concentration of the clear supernatant was evaluated using Bio-Rad Protein Assay Kit. Aliquots of equal amounts of proteins were subjected to SDS–PAGE. Proteins were then electrophoretically transferred to nitrocellulose membrane and non-specific sites were blocked with 5% skimmed milk in 1% Tween-20 (Sigma-Aldrich) in 20 mM TBS (pH 7.5) and reacted with the primary polyclonal antibody against cytochrome c and b-actin for 4 h at room temperature. After washing the tris-buffered saline containing 0.1% Tween-20, the membrane was then incubated with alkaline phosphatase-conjugated goat anti-rabbit secondary antibody. The protein bands were visualized using NBT–BCIP.

### Statistical analysis

GraphPadInstat software was used for statistical analysis. All data are expressed as the mean ± S.D. of three independent observations. The differences between the control and the treatment groups were determined by one-way analysis of variance (ANOVA). Post test was done using Dunnette Multiple Comparison Test to determine the significant levels.

## Results

### Antimutagenicity study by Ames test

Table 1 shows the results of antimutagenicity assays of the DTE (concentrations 1, 10, 100, 500, 1000 μg/plate) in *Salmonella* strains TA98 and TA100 by preincubation tests with metabolic activation. The antimutagenicity assays in preincubation tests showed significant antimutagenic effects of DTE, specially at higher concentrations, against the known positive mutagen B[a]P with S9 activation. In these assays the protective effects of DTE was found to be upto 58.6% and 44% on TA98 and TA100 respectively at the highest dose against B[a]P.

**Table 1:** **Antimutagenic effect of fractions Darjeeling tea extract (DTE) in Salmonella strains TA98 and TA100 in preincubation tests**

DTE (μg/plate)	Mean ± SD plus S9 mix
**TA 98**	
Control (100 μl of DMSO)	52 ± 12
B[a]P (50 ug/plate)	256 ± 23
B[a]P + 1	247 ± 21
B[a]P + 10	240 ± 18
B[a]P + 100	212 ± 20 **
B[a]P + 500	167 ± 16 **
B[a]P + 1000	106 ± 17 **
**TA 100**	
Control (100 μl of DMSO)	138 ± 12
B[a]P (50 μg/plate)	786 ± 34
B[a]P + 1	770 ± 25
B[a]P + 10	737 ± 21
B[a]P + 100	695 ± 36 *
B[a]P + 500	512 ± 24 **
B[a]P + 1000	440 ± 37 **

Each data represented as Mean ± SD of four plates. Results of each concentration of DTE plus positive mutagen benzo[*a*]pyrene (B[a]P) were compared with the positive mutagen treated group alone (all in presence of S9 mix) by Dunnett’s multiple comparison. *P < 0.05, **P < 0.01.

### Anticlastogenecity study by MN assay

Table 2 shows the results of anticlastogenic effects of four different concentrations (25, 50, 100, 200 μg/ml) of DTE against B[a]P as measured by micronuclei (MN) formation in human lymphocyte cultures. A statistically significant decrease in MN was observed in all the cultures of B[a]P post-treated with DTE at different concentrations when compared with the cultures that received B[a]P only. In MN assay the protective effect of DTE was found within a range of 16.4–56.8% in human lymphocyte (in vitro). The effect was maximum at 100 μg/ml concentration against the known positive mutagen B[a]P.

**Table 2:** **Anticlastogenic effect of Darjeeling tea extract (DTE) against benzo[a]pyrene (B[a]P) in human lymphocyte culture as measured by micronuclei formation**

Clastogen (μM)	DTE (μg/ml)	Number of micronucleated cells/1000 binucleated cells (mean + _S.D.)
-	25	1.33 ± 1.22
-	50	1.27 ± 0.98
-	100	1.35 ± 1.08
-	200	2.63 ± 1.46
B[a]P 100	-	18.69 ± 3.39
B[a]P 100	25	15.62 ± 2.37 *
B[a]P 100	50	12.56 ± 2.77 **
B[a]P 100	100	8.07 ± 1.68 **
B[a]P 100	200	10.49 ± 3.12 **

Each data represented as Mean ± SD of six samples per concentration. Results at of each concentration of DTE plus positive mutagen benzo[a]pyrene (B[a]P) were compared with the positive mutagen treated group alone (all in presence of S9 mix) by Dunnett’s multiple comparison. *P < 0.05, **P < 0.01.

### Cell viability assay using WST-1

**Figure 1:**
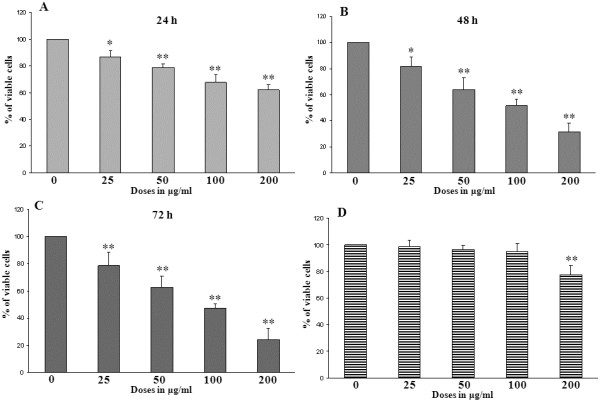
**Effects of Darjeeling tea extract (DTE) on the viability of U937 cells (A, B,C) and on peripheral blood mononuclear cells (PBMCs) (D) in the cell viability assay using WST-1 at different time points.** U937 cells (1 × 10^6^ per treatment) were treated with different concentrations (0, 25, 50, 100, 200 μg/ml) of DTE for 24 h **(A)**, 48 h **(B)** and 72 h **(C)** in triplicate. Similar experiment was performed on PBMCs but only 48 h treatment data is represented here **(D)**. The cell viability percentage at each concentration of treatment is plotted. The experimental results are represented as mean ± SD (n = 3) taking control (only vehicle i.e. 0.05% DMSO treated group) as 100% viable. *p < 0.05 and **p < 0.01 are the treated groups compared with control.

The effects of DTE on the viability of U937 cells were determined by obtaining the percentage of viable cells at varying concentrations (0, 25, 50, 100, 200 μg/ml) of DTE at different time points such as 24 h (Figure 1A), 48 h (Figure 1B) and 72 h (Figure 1C) of treatment respectively. The results obtained in this assay clearly shows that in each time points DTE have significant growth inhibitory effect on the selected cell line. The study also showed inhibition of U937 cell viability upon treatment with DTE which is both dose and time dependent. A similar experiment was performed on PBMCs (Data not shown for 24 h and 72 h of treatment). At above 100 μg/ml dose DTE showed toxicity towards PBMCs after 48 h of treatment (Figure 1D). Further experiments were conducted taking 100 μg/ml as highest concentration of DTE and the treatment time was optimized as 48 h.

### Induction of apoptosis in U937 cells upon treatment with DTE

**Figure 2:**
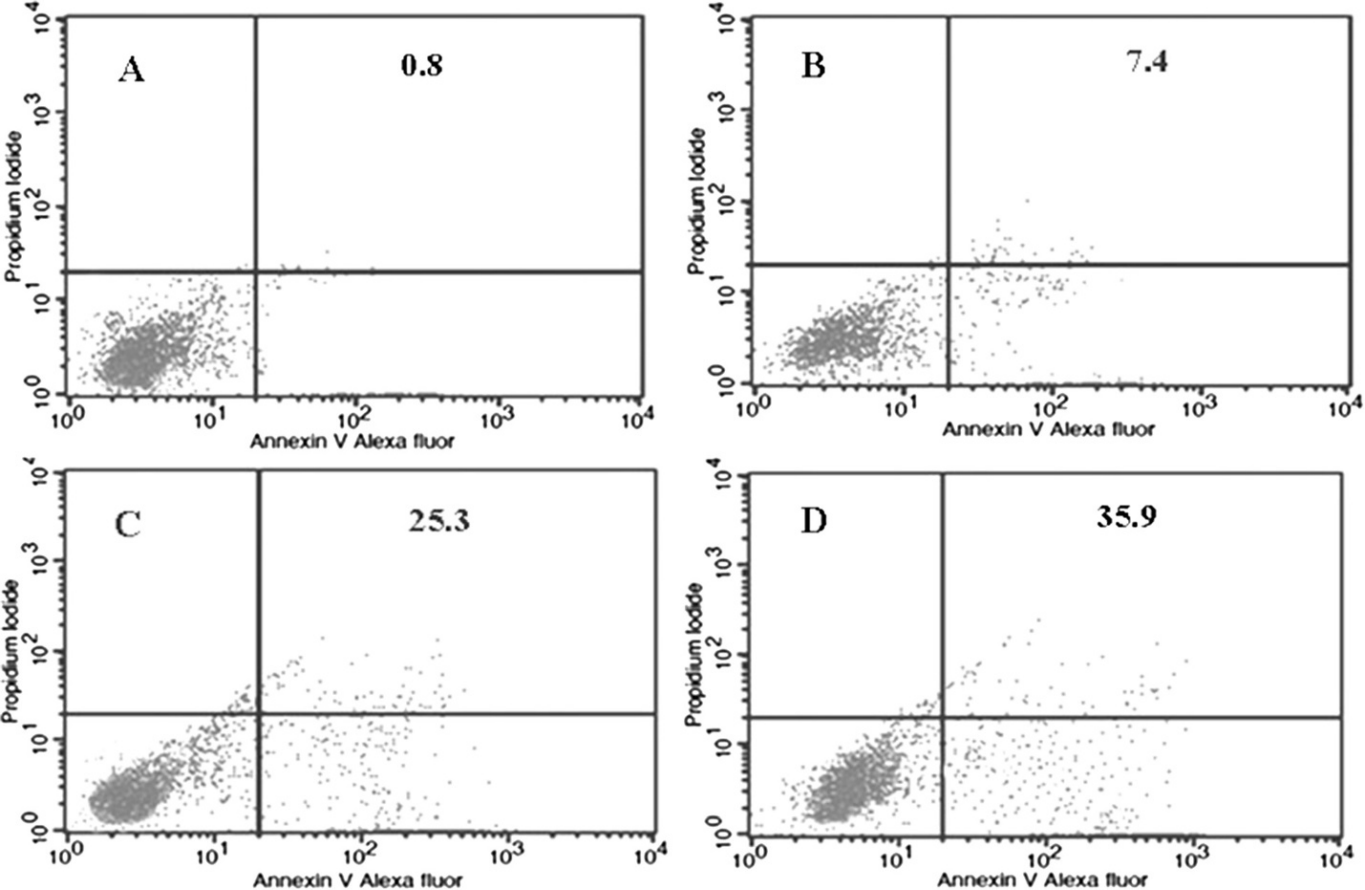
**Flow cytometric detection of apoptosis induction by three different doses of DTE at 48 h of treatment.** U937 cells (1 × 10^6^ per treatment) were incubated with DTE at 0 μg/ml **(A)**, 25 μg/ml **(B)**, 50 μg/ml **(C)** and 100 μg/ml **(D)** doses for 48 h. DTE induced apoptosis in U937 cells was determined by the flow cytometry using double staining method. Vehicle (0.05% DMSO only) treated control **(A)** and DTE treated cells **(B, C, D)** were labeled with PI and AnnexinV tagged-Alexa Fluor and then fixed and analyzed by flow cytometer. Dual parameter dot plot of Alexa fluor-fluorescence (X-axis) versus PI fluorescence (Y-axis) has been shown in logarithmic fluorescence intensity. Quadrants: lower left, live cells (- Alexa Fluor), lower right, apoptotic cells (+Alexa Fluor), upper right, necrotic cells or late phase of apoptotic cells (+PI, + Alexa Fluor). Numbers represented in each block here is the% of cells of lower right quadrant only.

Induction of apoptosis by DTE on U937 cells was studied by means of Annexin V/propidium iodide double staining method. The cells were treated with different concentrations of DTE (0, 25, 50, 100 μg/ml) for 48 h. and analysed by flow cytometry. The result indicates that the different doses of DTE (25, 50, 100 μg/ml) can induce apoptosis in U937 cells significantly and it ranged from 7.4% to 35.9% from lower to higher concentrations (Figure 2).

**Figure 3:**
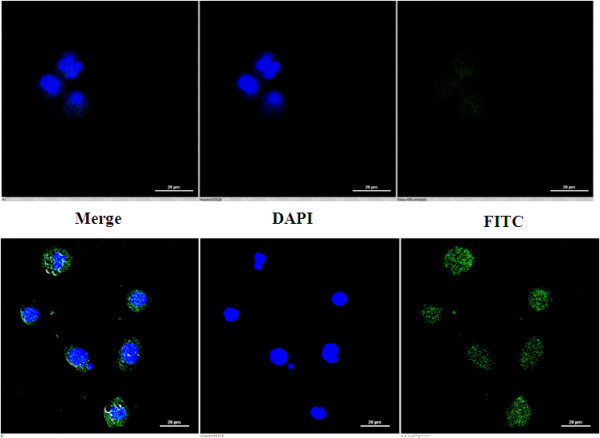
**Representative confocal pictures of DTE induced apoptotic cells.** U937 cells were incubated with DTE at 50 μg/ml dose for 48 h and after completion of stipulated time of treatment cells were labeled with FITC-AnnexinV (green fluorescence) and DAPI (blue fluorescence). The first row is showing cells treated with vehicle only (0.05% DMSO). The second row is showing treated cells with DTE. The first (extreme left) column showing merged (DAPI and FITC-AnnexinV) pictures, the second (middle) column showing only DAPI (blue) labeled cells, and the third column showing only FITC-tagged AnnexinV (green) labeled cells. Data shown here are from a representative experiment repeated thrice with similar results.

The induction of apoptosis by DTE was also evident from the confocal microscopic study. The DTE treated cells showed typical apoptotic feature i.e. externalization of phosphatidylserine on the outer surface of the treated cells. Only a representative picture has been incorporated here (Figure 3).

### Effect of DTE on caspase-3 activity

**Figure 4:**
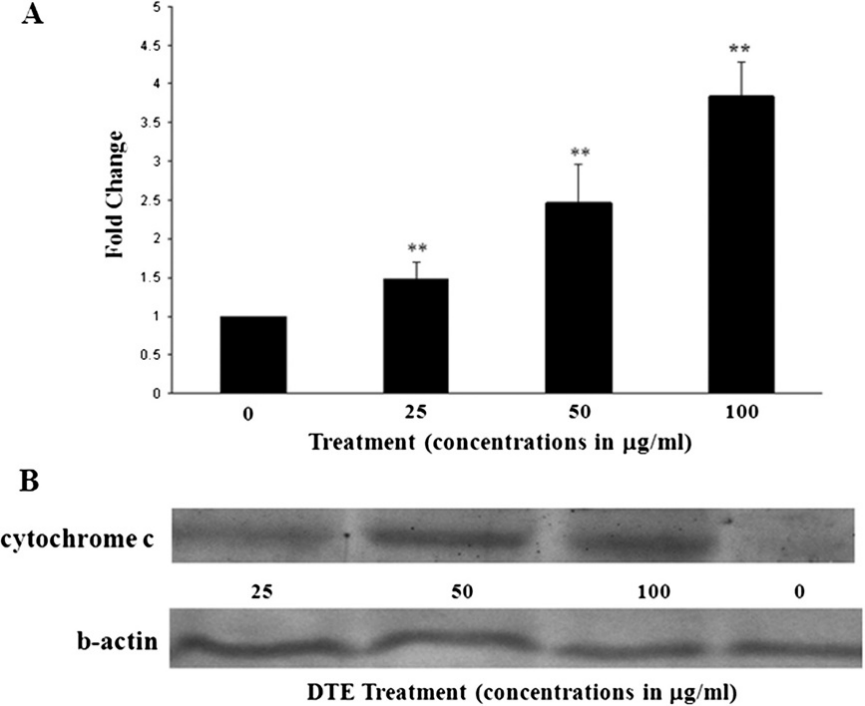
**Activation of caspase-3 and release of cytochrome c upon DTE treatment. A**: Caspase-3 activation after treatment with three different concentrations of DTE in U937 cells. Cells were treated with 0, 25, 50 and 100 μg/ml of DTE for 48 h and then subjected to measure caspase-3 activity by means of cleavage of the substrate DEVD-AFC. Each value was calculated as mean ± S.D. (n = 3). Bar diagram represents fold change in caspase-3 activity taking vehicle control as 1 fold. **B**: Release of cytochrome c in cytosolic fraction of U937 cells after treatment with DTE in dose-dependent manner. Cells were treated with DTE (0, 25, 50, 100 ug/ml) for 48 h. and were harvested and cytochrome c level in the cytosolic fraction was determined by western blot. Protein from cytosolic fraction was subjected to SDS–PAGE and western blot using cytochrome c and beta-actin antibody. Representative blot from three independent experiments was with identical results. Relative intensity of each band after normalization with the intensity of beta-actin in a blot (below) was measured.

Activation of caspases is a hallmark sign of apoptotic cell death. Among the caspase group caspase-3 is one of the most common and important caspase protease that promotes the execution phase of apoptosis. We examined whether this specific caspase was involved in DTE induced death of U937 cells. The activity of caspase-3 was examined after 48 h of treatment at different concentrations (0, 25, 50, 100 μg/ml). All fractions induced statistically significant (p < 0.05 to p < 0.01) increase of the activity of caspase-3 in U937 cells upon DTE treatment (Figure 4A).

### Cytochrome c release in the cytosol

To confirm the result further, we investigated the release of cytochrome c in cytosolic fraction of U937 cells to detect apoptosis. Western blot analysis showed that cytochrome c appeared in the cytosolic fractions after 48 h of incubation with DTE in a dose dependent manner (Figure 4B).

## Discussions

Ongoing research for the last few decades have shown that tea and its polyphenolic constituents can bring about many protective biological activities including antimutagenic and anticancer activities and consequently, tea appears to be a good natural agent for chemoprevention. Sufficient data have been generated asserting that preventive effects of green tea consumption on occurrence of various types of cancer. Black tea and its polyphenols have potentials to protect human beings from different detrimental health effect. Earlier reports are available on the antimutagenic potential of black tea or its extracts on bacterial system [5].

The main objective of this study was to investigate the antimutagenic and/or anticancer activities of Darjeeling tea extract (DTE). In this study, at first the mutagenic effects of DTE on bacterial system was monitored and no significant mutagenic effect was observed in the concentrations tested for antimutagenicity assay (data not shown). All the solvent control data and the mutagenicity assay data were with the normal range as mentioned by Maron and Ames [22]. Our experimental data indicates that DTE exhibits protective effects in vitro on bacterial system especially at the higher concentrations against the known mutagen B[a]P in presence of S9 mix. Result of this experiment was in agreement with a previous study where black tea polyphenols theaflavins and thearubigins showed significant antimutagenic activity against the same mutagen on bacterial systems [26].

To validate the data further the antimutagenic potential of DTE was tested in mammalian cell culture system using the same mutagen in presence of S9 mix. This assay result corroborates with the previous finding by showing less number of micronuclei formed in presence of DTE at different concentrations when compared with the mutagen B[a]P treated group only.

Development of cancer is often related with multiple mutations and hence it can be hypothesised that DTE could have anticancer activity as it was found to be a potent antimutagenic substance. Earlier it has been reported that tea polyphenols having antimutagenic potential also have anticancer effects [27]. Therefore, we subsequently tried to evaluate the anticancer effect of DTE on cancerous U937 cells. The objective of cell viability assay was to assess the direct growth inhibitory effect of the DTE on the proliferation and survival of the U937 cells [28]. Our results depicted inhibitory effects of DTE on the U937 cells at different time point. Decrease in the percentage of viable cells over a range of concentrations (0, 25, 50, 100, 200 μg/ml) of DTE at 24, 48 and 72 hours of treatment was observed. The decrease in the percentage of viable cells upon DTE treatment was found to be dose dependent.

"Apoptosis" is a type of programmed cell death which plays a very significant role in eliminating the hyperproliferating cells from our body. Induction of apoptosis in cancer cells, thus, can be considered as a protective mechanism against development and progression of this disease. In this study, we hypothesised that the loss of cell viability of U937 cells upon treatment with DTE could be due to the induction of apoptosis by the extract. Hence, we extended our study to find out the apoptosis induction by the extract. It has been observed that treatment with DTE resulted apoptosis in U937 cells as evident from Annexin V/propidium iodide double staining method. This result suggests that the inhibition of cell viability could be due to induction of apoptotic death of U937 cells upon DTE treatment. The confocal microscopy result also showed externalization of phosphotydle serine on the surface of DTE treated U937 cells which support the earlier evident of apoptosis induction.

Activity of caspase-3 is in many cases related with the apoptosis induction in cancer cells and an increase in the activity of this proteolytic enzyme is considered as a signature of the executionery phase of this process [29]. Here we found that, DTE, at all selected concentrations, was able to upregulate the activity of caspase-3 upon treatment in U937 cells. Another classical feature of apoptotic death is release of mitochondrial cytochrome c in cytosol [30]. We also investigated this phenomenon on U937 cells after DTE treatment. It was found that in almost all concentrations DTE was able to release cytochrome c in cytosol of U937 cells at 48 hours of treatment further proving the fact that DTE was able to induce apoptosis in the selected cancer cell line.

## Conclusion

Hence, this study demonstrated the antimutagenic and anticancer activities of the Darjeeling tea extract. DTE showed antimutagenic property on *Salmonella* strains in bacterial mutagenicity assay and on human lymphocytes in vitro with metabolic activation. DTE also showed inhibitory effect on the viability of the U937 cells. We further found that DTE exerts its antiproliferative effect by inducing apoptotic death in U937 cells. Therefore, it could be concluded that DTE have anticancer effects and could have chemopreventive properties.

## Abbreviations

WST-1: 2-(4-Iodophenyl)-3-(4-nitrophenyl)-5-(2,4-disulfophenyl)-2H-tetrazolium
B[a]P: Benzo[a]pyrene
DTE: Darjeeling Tea Extract
DTT: Dithiothreitol
DMSO: dimethyl sulfoxide
MN: Micronuclei
NADP: Nicotinamide adenine dinucleotide phosphate.
